# Flexible arthroscopy improves anterior visualization of the capitellum compared with a conventional 30° arthroscope: a cadaveric study

**DOI:** 10.1186/s13018-026-06940-y

**Published:** 2026-05-12

**Authors:** Maria Elze, Tobias Gruber, Stephanie Geyer, Kathi Thiele, Michael Hackl, Kilian Wegmann, Tim Leschinger

**Affiliations:** 1 Department of Orthopaedic, Trauma and Plastic Surgery, University Hospital Leipzig, Liebigstraße 20, 04103 Leipzig, Germany; 2 Faculty of Medicine and University Hospital, Center for Orthopedic and Trauma Surgery, University Hospital, Cologne, Germany; 3 Department for Shoulder and Elbow Surgery, St. Vinzenz Klinik Pfronten, Pfronten, Germany; 4 Department of Shoulder and Elbow Surgery, Center for Musculoskeletal Surgery Charite, Auguste Viktoria Hospital, Berlin, Berlin, Germany; 5 Department of Orthopaedic and Trauma Surgery, Medical Faculty Mannheim, University Medical Centre Mannheim, University of Heidelberg, Mannheim, Germany; 6 OCM (Orthopädische Chirurgie München) Clinic, Munich, Germany

**Keywords:** Elbow arthroscopy, Flexible optics, Capitellum visualization, Cadaveric elbow study

## Abstract

**Background:**

Arthroscopy of the elbow joint is usually performed with a 30° view. Stability tests, structural assessments and pathologies are described with this scope. Due to the anatomy of the distal humerus and the arthroscopic accessibility, the assessment of the capitellum, particularly with regard to cartilage damage, is limited in terms of dimension and depth. Arthroscopy with flexible optics in the range of 15° to 90° has not been routinely performed. The aim of the study was to investigate whether the use of flexible optics offers advantages in the assessment of the capitellum.

**Methods:**

Eleven fresh-frozen human cadaveric elbows were examined in a standardized free-hanging position with the elbow flexed to 90°. Arthroscopy was performed through the proximal posterolateral portal using a rigid 30° arthroscope and a flexible 15°–90° arthroscope. The most anterior visible margin of the capitellar articular surface was identified and marked arthroscopically for each optic. Following open surgical dislocation of the elbow, the distance between the two markings was measured in millimeters. Paired comparisons were performed using a paired t-test, and effect size was calculated using Cohen’s d.

**Results:**

In all specimens, the flexible 15°–90° arthroscope provided greater anterior visualization of the capitellum compared with the 30° arthroscope. The mean additional visible distance was 7.0 ± 2.5 mm (95% CI 5.3–8.7 mm). This difference was statistically significant (*p* < 0.001) with a large effect size (Cohen’s d = 2.654).

**Conclusions:**

A flexible 15°–90° arthroscope significantly increased anterior visualization of the capitellum compared with a conventional 30° arthroscope in this cadaveric model. Whether this anatomical visualization gain translates into clinical diagnostic or therapeutic benefit requires further investigation.

## Background

Elbow arthroscopy is an established procedure for the diagnosis and treatment of a broad spectrum of intra-articular pathologies, including osteochondral lesions, loose bodies, synovitis, and post-traumatic conditions. Standardized portal placement and systematic diagnostic routines are well described, and a rigid 30° arthroscope remains the most commonly used optic in elbow arthroscopy [1, 2]. This optic provides an adequate field of view and depth perception for most compartments of the joint and has therefore become the standard in clinical practice.

Despite these advantages, visualization of the capitellum remains technically challenging. The convex geometry of the capitellum, its predominantly anterior orientation, and the confined anatomy of the radiocapitellar joint restrict the field of view obtainable through standard posterolateral portals [3]. Even with optimized portal placement and varying degrees of elbow flexion, complete visualization of the anterior articular surface is often limited. Consequently, chondral lesions—particularly those located at the anterior or anterolateral aspect of the capitellum—may be partially visualized or underestimated during diagnostic arthroscopy. This limited accessibility represents a clinically relevant diagnostic challenge because several common capitellar pathologies, including osteochondritis dissecans and focal traumatic chondral defects, frequently involve the anterior articular surface. Even under standardized posterolateral viewing conditions, the surgeon may therefore be unable to fully appreciate lesion extension, cartilage stability, or exact defect boundaries using a conventional 30° optic alone.

To address similar anatomical limitations in other joints, arthroscopes with steeper viewing angles have been introduced. A 70° optic has been shown to improve visualization in hip [4] and knee arthroscopy [5] and has also been applied in selected elbow procedures [6]. Increasing the viewing angle can expand the visualized articular surface; however, fixed steep-angle optics may be associated with image distortion, altered spatial orientation, and a steeper learning curve. As a result, they have not become routine in elbow arthroscopy.

Flexible arthroscopes with adjustable viewing angles, typically ranging from 15° to 90°, offer a potential alternative. Unlike fixed-angle optics, flexible systems allow continuous adaptation of the viewing direction to the individual joint anatomy without requiring portal changes. This adaptability may facilitate improved visualization of anatomically difficult-to-access regions, such as the anterior capitellum, while maintaining orientation within the joint. Although flexible arthroscopy has been described in other joints [7], quantitative data evaluating its benefit for capitellar visualization in the elbow are currently lacking.

Therefore, the purpose of this cadaveric study was to quantitatively compare anterior visualization of the capitellum using a standard rigid 30° arthroscope and a flexible 15°–90° arthroscope through the proximal posterolateral portal. We hypothesized that the flexible optic would allow significantly greater anterior visualization of the capitellum compared with the conventional 30° arthroscope.

## Methods

### Study design and specimens

Following institutional review board approval (ID 22-1451), eleven fresh-frozen human cadaveric elbows with intact soft tissue envelopes were included in this comparative cadaveric study. Specimens were excluded if macroscopic signs of previous trauma, advanced degenerative changes, or prior surgical procedures of the elbow joint were present. Donor demographics including age, sex, and laterality were recorded.

All specimens were thawed at room temperature for 24 h prior to testing and kept moist throughout the procedures to minimize tissue desiccation.

### Experimental setup and arthroscopic technique

**Fig. 1:**
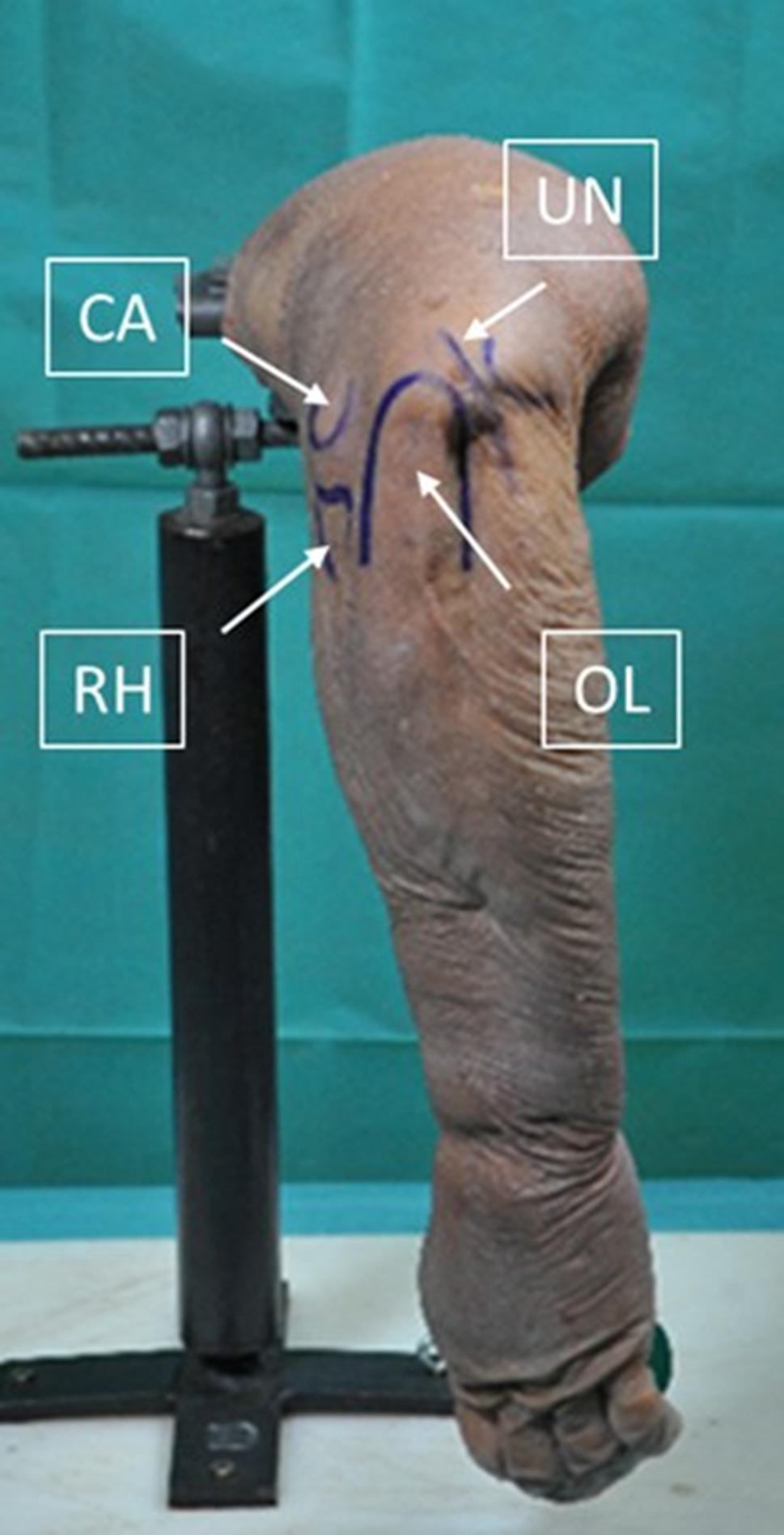
Anatomical landmarks for standard elbow arthroscopy. RH,  radial head; CA, capitellum; OL, olecranon; UN, ulnar nerve

**Fig. 2:**
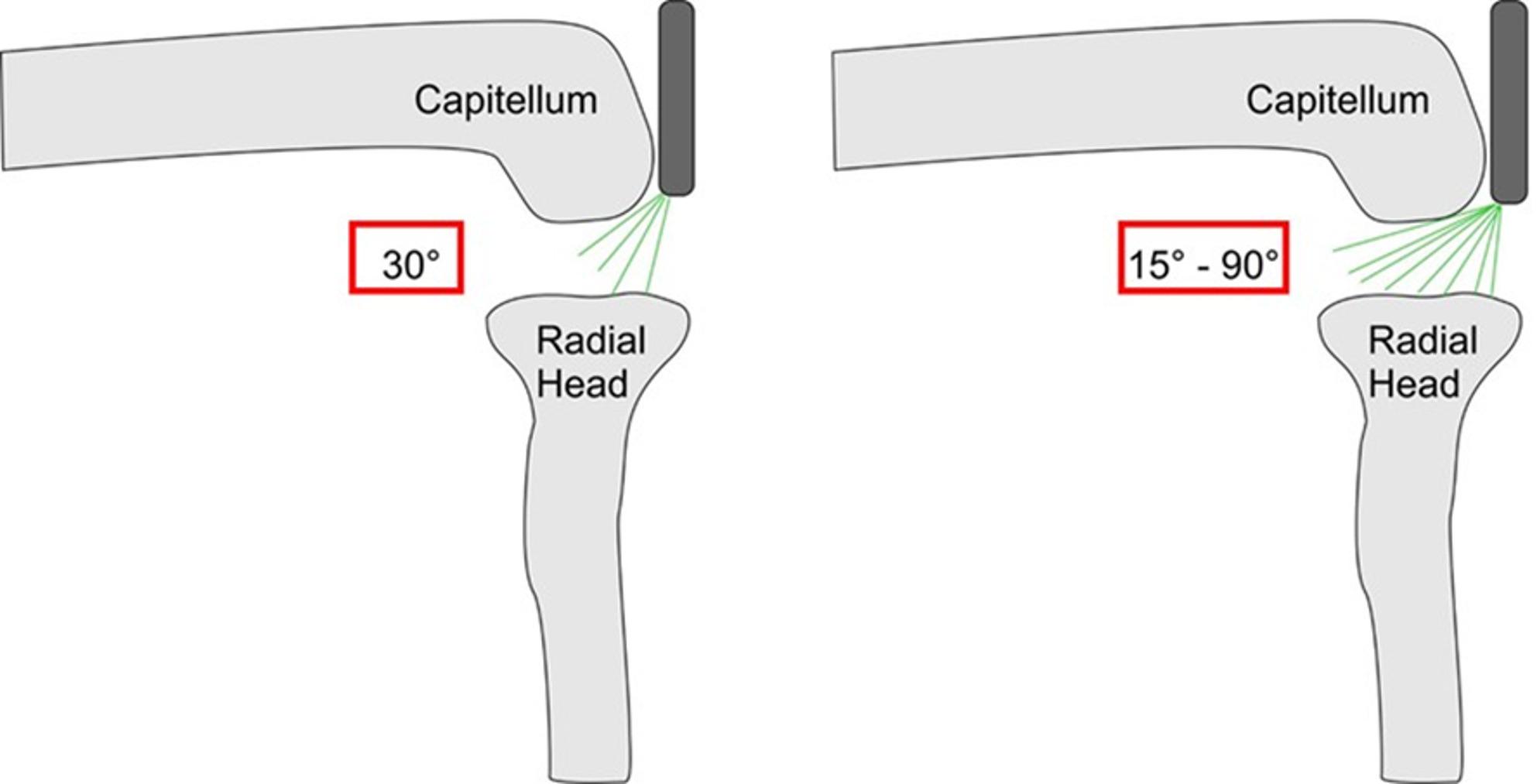
Comparison of rigid 30° and flexible 15°–90° arthroscopes through the high posterolateral portal. The flexible optic allows adjustable viewing angles for improved visualization of the anterior capitellum

Each specimen was fixed at the proximal humerus in a custom holding device, allowing free motion of the elbow joint. The elbow was positioned in 90° of flexion throughout the measurements. Bony landmarks were identified and marked, including the olecranon, radial head, and ulnar nerve (Fig. 1). Arthroscopy was performed through a proximal posterolateral viewing portal as previously described [8]. A distal posterolateral portal was used as a working portal for cartilage marking. Two arthroscopes were evaluated sequentially in each specimen: a rigid 30° arthroscope (Arthroscopy optic, 30°, 3 mm x 138 mm, Arthrex, Naples, USA) and a flexible 15°–90° arthroscope (Endocameleon Arthro Hopkins optic, Karl Storz, Tuttlingen, Germany) (Fig. 2). The order of optic use was alternated between specimens to minimize systematic bias. The proximal posterolateral portal at 90° of elbow flexion was deliberately selected because it represents a standardized and commonly used viewing configuration for assessment of the radiocapitellar joint while allowing reproducible comparison between the two optical systems. During sequential testing, the surgeon identified the maximally visible anterior cartilage boundary independently for each optic under direct arthroscopic visualization. Although a previous cartilage puncture mark was present, no continuous needle tract existed that could mechanically determine the subsequent marking position.

**Fig. 3:**
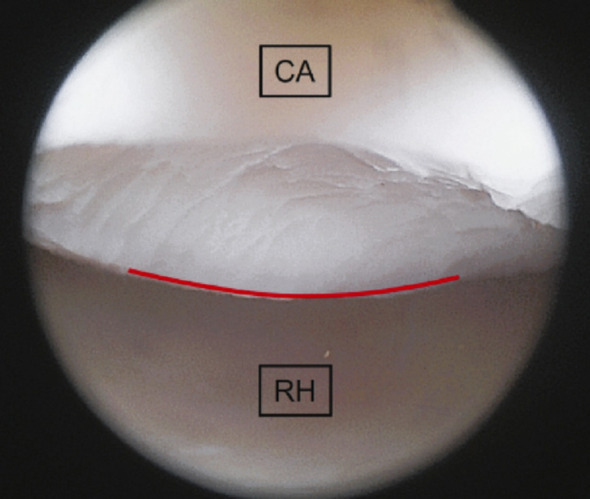
Marking of the maximally visible anterior portion of the capitellar cartilage using the rigid 30° arthroscope. Red solid line indicates the ventral visual boundary. RH, radial head; CA, capitellum

**Fig. 4:**
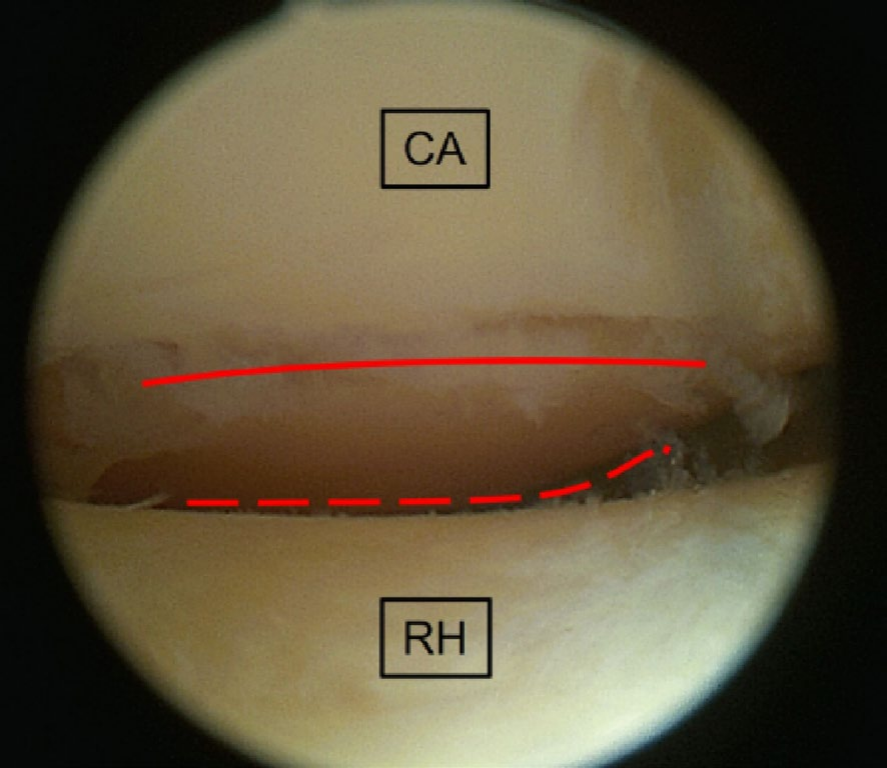
Marking of the maximally visible anterior portion of the capitellar cartilage using the flexible 15°–90° arthroscope (black dashed line). Comparison with the 30° arthroscope (red solid line) demonstrates increased anterior visualization. RH, radial head; CA, capitellum

Using each optic, the most anterior visible margin of the capitellar articular surface was identified arthroscopically. A sharp spinal needle introduced through the distal posterolateral portal was used to mark this margin directly on the cartilage surface. Care was taken to maintain identical portal positions and elbow flexion for both optics (Figs. 3 and 4).

### Open dislocation and measurement protocol

**Fig. 5:**
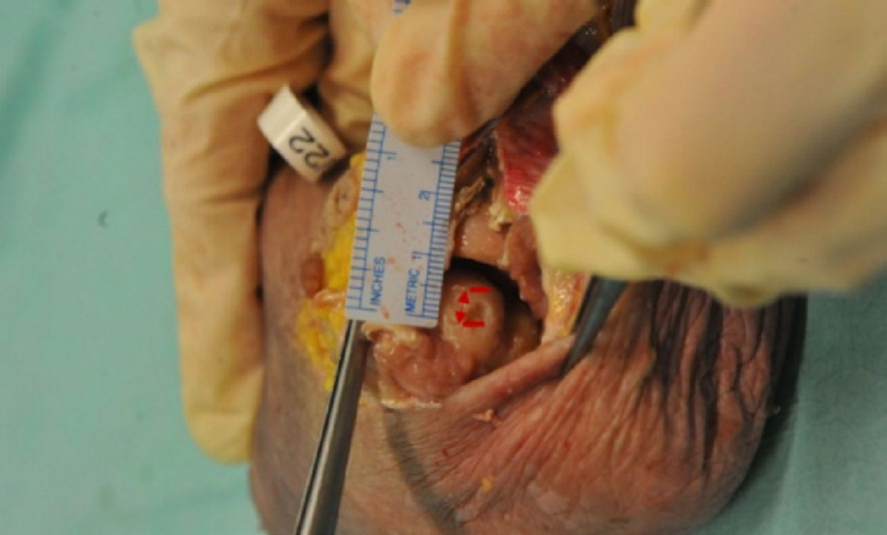
Open measurement of the distance between cartilage markings following surgical elbow dislocation. The distance corresponds to the additional anterior visualization achieved with the flexible 15°–90° arthroscope. Scale bar = 1 mm

After arthroscopic marking with both optics, an open surgical dislocation of the elbow joint was performed by releasing the radial ligamentous structures to fully expose the radiocapitellar joint. The distance between the two cartilage markings corresponding to the maximum anterior visualization achieved with each optic was measured using a calibrated millimeter ruler (Fig. 5). The measurement accuracy of the ruler was ± 0.5 mm.

All measurements were performed twice by the same observer, and the mean value was used for statistical analysis. Formal interobserver reliability testing was not performed because the study was designed as an initial exploratory anatomical comparison under maximally standardized conditions using one experienced examiner.

### Outcome measure

The primary outcome measure was the difference in millimeters between the anterior visualization achieved with the flexible 15°–90° arthroscope and the rigid 30° arthroscope.

### Statistical analysis

Statistical analysis was performed using SPSS software (version 29.0; IBM, Armonk, NY, USA). Data distribution was assessed visually and using the Shapiro–Wilk test. Paired comparisons between the two optics were performed using a paired t-test. Statistical significance was defined as *p* < 0.05. Effect size was calculated using Cohen’s d.

## Results

**Fig. 6:**
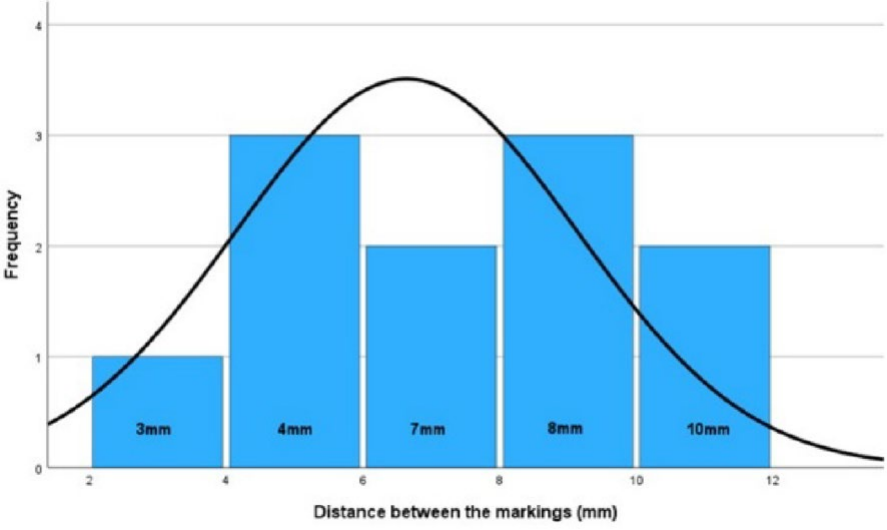
Distances between the markings under the 30° optic and the 15–90° optic

All eleven cadaveric elbow specimens were successfully examined according to the predefined protocol. Six right and five left elbows were included. The mean donor age was 87 ± 9 years (range, 76–99 years). In every specimen (11/11, 100%), the flexible 15°–90° arthroscope allowed visualization of a more anterior portion of the capitellar articular surface compared with the rigid 30° arthroscope when viewed through the proximal posterolateral portal at 90° of elbow flexion. The mean additional anterior visualization achieved with the flexible optic was 7.0 ± 2.5 mm (range, 3–10 mm). Paired comparison using a paired two-tailed t-test demonstrated a statistically significant difference between the two optics (mean difference 7.0 mm; 95% confidence interval [CI], 5.3–8.7 mm; *p* < 0.001). The calculated effect size was large (Cohen’s d = 2.654), indicating a substantial magnitude of difference between the visualization achieved with the two optical systems. Individual specimen data are summarized in Table 1. No specimen showed inferior visualization with the flexible optic compared to the 30° arthroscope (Fig. 6).

**Table 1:** Individual donor demographics and corresponding test results

Specimens number	Gender	Affected arm	Age in years	Distance between markings in mm
1	Male	Left	81	7
2	Female	Right	99	8
3	Female	Left	77	4
4	Male	Right	76	8
5	Female	Left	89	8
6	Female	Right	91	4
7	Male	Left	92	10
8	Female	Left	99	10
9	Female	Right	96	7
10	Female	Right	99	4
11	Female	Right	77	3

## Discussion

The principal finding of this cadaveric study was that flexible 15°–90° arthroscopy provided significantly greater anterior capitellar visualization than a conventional rigid 30° arthroscope when both were used through the proximal posterolateral portal at 90° of elbow flexion. Although this parameter reflects only a linear anatomical measurement, it provides an objective first quantification of the otherwise difficult-to-estimate visualization limitations associated with standard elbow arthroscopy optics.

To better contextualize the magnitude of the additional visualization observed in this study, previously reported anatomical dimensions of the capitellum should be considered. In a computed tomography–based morphometric analysis of 50 elbows, Giuseppe Giannicola and colleagues [9] reported a mean sagittal diameter of the capitellum of 10.7 mm (SD 1.1). The additional visualization of approximately 7 mm observed with the flexible arthroscope in the present study therefore corresponds to roughly two thirds of the reported anteroposterior dimension of the capitellar articular surface.

Considering the reported mean sagittal diameter of 10.7 mm, the observed additional visualization of approximately 7 mm suggests that a substantial portion of the previously inaccessible anterior capitellar surface may become assessable when flexible optics are used. Nevertheless, the present study did not quantify the exact percentage of the total articular surface visualized, nor did it evaluate lesion detectability or diagnostic sensitivity. Therefore, the observed anatomical gain should be interpreted as an experimental visualization advantage rather than as direct proof of superior clinical diagnostic performance.

Limited visualization of the capitellum during elbow arthroscopy has been recognized as a technical challenge in several previous investigations [3]. Studies evaluating the influence of portal placement have demonstrated that visualization of the radiocapitellar joint varies considerably depending on the chosen arthroscopic approach [10–12]. In addition, arthroscopes with steeper viewing angles have been used in other joints to overcome anatomical constraints and expand the visible articular surface.

Fixed high-angle optics, such as 70° arthroscopes, have been reported to improve visualization in hip and knee arthroscopy and have occasionally been used in elbow procedures [4, 13]. However, the abrupt change in viewing angle associated with such optics can make spatial orientation more difficult and may require a substantial learning curve for surgeons accustomed to conventional 30° arthroscopes [7]. A direct comparison with a rigid 70° arthroscope was not performed in the present study. Such a comparison would be of particular practical interest, as 70° optics already represent an established strategy to improve visualization in difficult arthroscopic regions. Consequently, it remains unclear whether flexible optics provide incremental benefit beyond currently available high-angle rigid systems.

The flexible arthroscope evaluated in the present study differs fundamentally from fixed-angle optics because it allows continuous adjustment of the viewing direction. This capability enables the surgeon to dynamically adapt the viewing angle to the specific joint anatomy without changing portals or instruments. The increased anterior visualization observed in this study may therefore be explained by the ability of the flexible optic to direct the field of view toward otherwise difficult-to-access regions of the capitellar surface.

While increasing the viewing angle can expand the visible articular surface, optical distortion is a known phenomenon in arthroscopy. Experimental studies have demonstrated that lens angle, portal position, and viewing direction can influence image distortion and depth perception [7, 14, 15]. Although flexible arthroscopes allow gradual modification of the viewing direction, high degrees of angulation may still be associated with optical distortion or altered spatial orientation. In addition, ergonomic handling, surgeon comfort, hand-eye coordination, and the possible influence on operative efficiency were not assessed in this cadaveric investigation. Increased anatomical visibility alone should therefore not be equated with improved intraoperative practicality.

Several limitations of this study must be acknowledged. First, the number of examined specimens was relatively small. However, because this was a paired comparative cadaveric study in which each elbow served as its own control, interspecimen anatomical variability was reduced and a direct internal comparison between optical systems was possible. Still, the limited sample size restricts external generalizability.

Second, the cadaveric donors were of advanced age. Degenerative cartilage changes and age-related alterations in joint morphology may differ from the anatomy typically encountered in younger patients with capitellar osteochondral lesions, thereby limiting direct clinical transferability.

Third, only one portal configuration and one elbow position were evaluated. Visualization was assessed exclusively through the proximal posterolateral portal at 90° of elbow flexion while forearm rotation was kept constant. Previous studies have shown that portal placement, elbow flexion angle, and forearm positioning may significantly influence radiocapitellar accessibility [13]. Therefore, the isolated effect of flexible optics under alternative dynamic arthroscopic conditions remains unknown.

Fourth, measurements were performed by a single experienced observer, and formal interobserver reliability was not assessed. Although all measurements were repeated twice and averaged, observer-dependent bias cannot be completely excluded. In addition, complete blinding to the previous cartilage marking during sequential optic testing was not technically feasible.

Fifth, no rigid 70° arthroscope was included as an additional comparator. Therefore, the relative benefit of flexible optics compared with other currently available high-angle arthroscopic systems remains to be determined.

Finally, the present study focused exclusively on linear additional visualization distance. Neither the percentage of the total capitellar surface visualized nor lesion detection rates, diagnostic accuracy, image distortion, or ergonomic handling characteristics were assessed. Consequently, direct clinical superiority of flexible arthroscopy cannot be concluded from the present experimental findings alone.

Future investigations should evaluate flexible arthroscopy under dynamic intraoperative conditions and include different elbow flexion angles and portal configurations. Quantification of the proportion of the capitellar articular surface visualized with different optical systems may further clarify the practical relevance of flexible arthroscopy. Most importantly, prospective clinical studies will be required to determine whether the improved visualization observed in this experimental setting translates into enhanced diagnostic accuracy or improved surgical outcomes.

## Conclusion

In this cadaveric study, the use of a flexible 15°–90° arthroscope significantly increased anterior visualization of the capitellum compared with a conventional rigid 30° arthroscope when applied through the proximal posterolateral portal at 90° of elbow flexion.

The flexible optic consistently allowed visualization of a more anterior portion of the articular surface under standardized experimental conditions. This finding indicates a measurable anatomical visualization advantage; however, no direct conclusions can yet be drawn regarding improved lesion detection, operative efficiency, or superior clinical outcomes.

Further comparative studies including alternative portal strategies, high-angle rigid optics, and prospective clinical validation are warranted before routine clinical adoption can be recommended.

## Data Availability

All data generated or analysed during this study are included in this published article.
